# On the Epidemic Catarrh Commonly Termed Influenza

**Published:** 1804-06-01

**Authors:** John Moodie


### *On the* Epidemic Catarrh *commonly termed* Influenza.

By John Moodie, M.D.

Much has already been written on this disorder; however, the following observations, although the result of my own reflections on the subject, are not therefore offered to the public in support of any particular theory; considering it a duty incumbent upon every medical practitioner to contribute to the utmost of his power to the stock of general information. Having recently seen several cases of well marked influenza in this city, some distinct, others more or less complicated with the prevailing complaints of the season; in order to ascertain how nearly the symptoms resembled those of the last year, I attentively observed the progress and termination of the disorder, especially when unaccompanied with other diseases, and found no material difference, nor was any other method of treatment necessary.

Through the medium of the public prints, the community at large were for some time much alarmed by an apprehension of this epidemic; and the unfavorable impression tending to remove confidence from the mind, occasioned a despondency, which, if any contagion really existed, must have been conducive to its more extensive dissemination. Hence, among the lower orders of society, the dread of infection operating to the disadvantage of the afflicted, the offices of the friend, and of the nurse, may have been with-held from an ill grounded fear of danger.

On

On the public mind, the first impression is too often the strongest, and the most lasting. To do away that impression before it shall sink too deep, is the laudable intention of the following remarks, which, after what has been said by others on the subject, may be found in a great measure unnecessary. Yet they may tend to shew, that notwithstanding what has hitherto been asserted respecting the nature and cause of this disorder, nothing conclusive has been advanced by any writer in favour of contagion. The great questions are well known to consist in, whether the epidemic be contagious? and whether it be an imported disease?

The variety of opinions prevailing among speculative men, in what relates to the origin and cure of the late epidemic catarrh, commonly termed Influenza, as well as to what regards the origin and cure of the plague, and some other diseases, is a convincing proof of the fallibility of the human mind. The subject of physic is attended with so many difficulties, that we frequently depend upon conjecture rather than certain facts; and this will always be the case in every science where so little can be decided by demonstration and actual experiments, and where the rest depends upon the caprice of our reasoning faculties, which are often insensibly perverted by the early prejudices of education and other causes. Things thus seen through various mediums, must necessarily strike our senses very differently, though in their nature they are essentially the same.

Observations, therefore, founded upon experience are the surest guide to truth in every science; and when applied to the prevention and cure of diseases, are alone more likely to succeed than the most refined and plausible theories where these are disregarded and set aside.

In the profession of Physic, where the practitioners are considered as the guardians of health, the community at large have a claim upon their exertions; hence moral duty should stimulate them to offer every information that may tend to universal good. It is beneath the dignity of a physician to consider himself the object of his own attention; he should deal out his bounty with a liberal hand, and let his discoveries be diffusive.

It appears from the description of epidemic diseases transmitted to us by eminent physicians, that a disorder resembling that which we now term *influenza* prevailed at different periods in various parts of the world, from 1510 to the present time. And although the diseases which form form the prevailing epidemic may not have formerly assumed the characteristic symptoms which they have lately done; yet this is probably owing to particular circumstances, arising chiefly from the temperature of the seasons when they prevailed. It is, however, remarkable, that during the last century, when the plague so frequently visited England, the influenza appeared only once, viz. in 1657; whereas it has prevailed among us no less than seven times in the space of nearly eighty years.

Sir John Pringle was of opinion, that the sensible qualities of the atmosphere had no effect in producing the epidemic disease prevalent in London, and almost the whole kingdom, in the autumn of 1775. He also thinks that such diseases do not depend upon any principles with which we are acquainted; but upon some others, to be investigated by the united enquiries of scientific men.

However, if I may be permitted, from my own observation, to form any reasonable conclusions, I cannot but attribute the proximate cause of the epidemic catarrh, which appeared in the latter end of 1792, to the uncommonly hot, moist, and heavy temperature of the atmosphere, during the summer of that year. For it is a fact well known, that the vicissitudes of heat and moisture are extremely injurious to the human frame, especially when they rapidly succeed each other. Hence the great unwholesomeness of sudden calms, or heavy rains, after long droughts; and of sudden thaw after severe frosts. It is thus that the constitution of the atmosphere, which cannot be doubted, considering its great influence on valetudinary habits, shews itself; by which the human body is more or less disposed to diseases in general at one time than another, but particularly colds, the small-pox, epidemic dysentery, and ulcerated sore throat; whence diseases of the putrid kind not only become more frequent, but also more fatal, and are known to prevail with greater violence, as the air changes from a healthy to a malignant state.

The great plague in London, in the year 1636, which lasted twelve years, was more or less fatal at different periods. In eight years, one with another, two thousand people died yearly, and never less than eight hundred in one year; which shews that contagion and its mortal effects, depended as much upon the atmosphere as the disease itself. This circumstance is still more clearly proved, by the great disproportion of deaths in different weeks; the number in one week increasing from one hundred and

 eighteen eighteen to nine hundred and twenty-seven in the next; and in another, decreasing from nine hundred and ninety-three to two hundred and fifty-eight; and from that number again increasing, the next week, to eight hundred and fifty-two.*

In what manner diseases are occasioned or influenced by the obvious qualities of the air, is difficult to determine; notwithstanding all that has been said on its gravity and lightness, effects of heat and cold, moisture and dryness, or the winds blowing from particular quarters at certain seasons, with different degrees of violence; seeing that very sudden changes of weather from one extreme to another, frequently happen without producing any diseases of a malignant or epidemic nature.

Dr. Mead remarks, that contagion is propagated by three causes, the air, diseased persons, and goods transported from infected places.

Ancient authors, who lived in countries more exposed to these calamities than our own, observed the constitution of the atmosphere, which preceded pestilential fevers, to be great heats, attended with much rain and southerly winds; and it is an opinion as old as *Hippocrates*, corroborated by almost every subsequent writer who has treated on the subject, that no other than a hot and moist temperature of the air brings on the plague; and that the duration of this constitution is the measure of the violence of the distemper.

Whatever the temperature of the seasons in this quarter of the globe might have been a century ago, it is evident that the climate of England, and perhaps of other parts of the world, has undergone a very considerable alteration during the last thirty years, and is now become more changeable, moist and cold, than before.

The epidemic diseases which have at former periods, and recently prevailed in this country, may therefore be referred to the Spring and Autumn, when the particular state of the air has been unusually hot and moist, and subject to sudden changes. Hence the effusion of a general existing cause, rendering diseases epidemical, though not infectious, from one person to another, yet existing where we may be said to be living in an atmosphere more or less impregnated with contagion, according to the locality of the situation.

We

* Grant on the Bills of Mortality.

We have therefore every reason to suppose, that the prevailing complaints have arisen from the sudden changes of the weather which have lately taken place. From hence it would seem, that the infection must depend upon some powerful operating principle, by which means certain noxious effluvia are absorbed from the air into the system generally acting as a morbid cause.

But whether the disorder is, or is not contagious, is a fact which, perhaps, still remains to be ascertained, and sanctioned by the most respectable authority.—Nevertheless, it is certain that whole families in this city, as well as in various and remote parts of the country, have, on the same day, been indiscriminately seized with the complaint, where there had not been any previous communication with those already affected. Neither were the medical attendants, nor others, in any respect liable to infection. During the prevalence of the disease, I knew persons who came hither from different parts of the kingdom, with a view (as they said) to avoid the disorder, but actually brought it with them, which was not to my knowledge, communicated to others who resided in the same house.

Catarrhal affections, which may be properly termed well-marked influenza, as far as my practice and information extended, first made their appearance in this city and neighbourhood about the beginning of November, 1802, and did not entirely disappear until near the middle of June. About this time, I was desired to visit a child a year and a half old, who had hitherto enjoyed good health, but died of the catarrhus after a short illness, unaccompanied with any other species of the epidemic.

*Bath, April* 10, 1804.

[ To be continued. ]

